# Feasibility and utility of a multi-segment foot model to measure joint kinematics in older aged adults during gait

**DOI:** 10.1186/1757-1146-5-S1-P1

**Published:** 2012-04-10

**Authors:** John Arnold, Shylie Mackintosh, Sara Jones, Dominic Thewlis

**Affiliations:** 1 School of Health Sciences, University of South Australia, Adelaide, South Australia, 5000, Australia

## Background

Multi-segment foot models (MSFMs) have been developed for the representation of foot kinematics [1,2]. Despite the existence of models for different populations there is a paucity of information about the feasibility and utility of these models in older aged individuals. As both morphological and functional differences of the feet exist between different age groups, it is important to establish if it is feasible to apply the models developed for younger age groups in older individuals.

## Materials and methods

Participants were individuals aged 50 to 90 years who could ambulate independently and were free of any known neurological or musculoskeletal disorders. A five segment foot and ankle model was selected for use in this study [3]. Five walking trials were collected from each participant. Kinematic and ground reaction force data were collected with twelve optoelectronic cameras (FLEX:V100R2, OptiTrack, Natural Point Inc., Oregon, USA) and two force platforms (Kistler Instrument Corp, Switzerland) at 100 Hz and 400 Hz respectively. Data were exported to Visual3D v4.0 for analysis (C-Motion Inc., MD, USA).

Marker placement reliability (intra and inter-rater) was assessed by two raters applying markers on two occasions. The propagation of differences in marker placement to stance phase joint kinematics was explored, including evaluation of model repeatability.

## Results and conclusions

Data collection for the study will be finalised in January 2012. Statistical analysis of the data will be performed with the findings prepared for presentation. It is anticipated that the results of this study will provide preliminary evidence to justify the selection of this foot model for use in older aged individuals.
